# DNA Damage Inducible Transcript 4 Gene: The Switch of the Metabolism as Potential Target in Cancer

**DOI:** 10.3389/fonc.2018.00106

**Published:** 2018-04-12

**Authors:** Indira Tirado-Hurtado, Williams Fajardo, Joseph A. Pinto

**Affiliations:** ^1^Unidad de Investigación Básica y Traslacional, Oncosalud-AUNA, Lima, Peru; ^2^Escuela de Medicina Humana, Universidad Privada San Juan Bautista, Lima, Peru

**Keywords:** DNA damage inducible transcript 4, mammalian target of rapamycin, malignant tumors, biomarkers, targeted therapies

## Abstract

DNA damage inducible transcript 4 (*DDIT4*) gene is expressed under stress situations turning off the metabolic activity triggered by the mammalian target of rapamycin (mTOR). Several *in vitro* and *in vivo* works have demonstrated the ability of *DDIT4* to generate resistance to cancer therapy. The link between the metabolism suppression and aggressiveness features of cancer cells remains poorly understood since anti-mTOR agents who are part of the repertoire of drugs used for systemic treatment of cancer achieving variable results. Interestingly, the high *DDIT4* expression is associated with worse outcomes compared to tumors with low *DDIT4* expression, seen in a wide variety of solid and hematological tumors, which suggests the driver role of this gene and provide the basis to target it as part of a new therapeutic strategy. In this review, we highlight our current knowledge about the biology of *DDIT4* and its role as a prognostic biomarker, encompassing the motives for the development of target drugs against *DDIT4* as a better target than mTOR inhibitors.

## Background

Cancer is a complex disease arising from the gradual accumulation of genetic changes resulting in the reprogramming of key cellular processes well described in “The Hallmarks of Cancer” written by Hanahan and Weinberg (1). Structural and functional alterations in driver genes and entire pathways to fulfill the nutritional requirements are responsible for this reprogramming, and although their mechanisms are not fully known, various drugs have been developed to target actionable mutations (2, 3).

One attractive therapeutic strategy is the inhibition of the mammalian target of rapamycin (mTOR), as well as various downstream and upstream signaling molecules (4). The mTOR pathway has evolved as nutrient sensing to promote cell proliferation under adequate nutritional and environmental conditions (5). The activation of mTOR depends on the formation of two complexes called mTOR complex 1 (mTORC1) and mTOR complex 2 (mTORC2) that are activated in distinct ways. mTORC1 controls the protein synthesis and cell survival through the phosphorylation of its substrates, 4EBP1, p70S6K and factor 4G. mTORC1 is inactivated by rapamycin but is activated by growth factors, nutrients, energy and stress signals, and essential signaling pathways (PI3K, MAPK, and AMPK). In contrast to mTORC1, mTORC2 is not inactivated by rapamycin and generally, it is not affected by nutrients and energy signals. mTORC2 regulates cytoskeleton organization and promotes cell survival through the phosphorylation of protein kinase B (Akt) and protein kinase C (6–8). The key role of mTOR in these processes explains its association in pathologies such as cancer (9, 10).

Several works and recent cancer genomic projects described high rates of mutations in genes involved in the mTOR pathway, including *PI3K, PTEN, AKT*, and *S6K1, 4EBP1*, and *EIF4E*. Based in this data, mTOR inhibitors (rapamycin and its analogs) have become attractive therapeutic agents tested in several clinical trials, as single agents or in combination with other types of systemic treatment (9). Unfortunately, some inhibitors of mTOR have shown lack of efficacy, including the rapamycin (11).

The DNA damage inducible transcript 4 (*DDIT4*) is induced by cellular stress conditions and regulates the mTOR activity (12), and also its abnormal expression has been linked to multiple diseases, including malignant tumors (13–18).

## *DDIT4* Gene

This gene was simultaneously discovered and cloned in 2002 by two independent research groups. Shoshani et al. using a microarray hybridization technique to investigate the hypoxia-dependent gene expression in rat glioma C6 cells reported a gene highly upregulated in response to hypoxia-inducible factor 1 (HIF-1) and regulating the generation of cellular reactive oxygen species (ROS). This gene was designated *RTP801* (19). Concurrently, Ellisen et al. identified a new p53 target induced by DNA damage and regulated by p63 during embryogenesis and epidermal differentiation. In this study, this gene was alternatively designated *REDD1* (regulated in development and DNA damage response 1), which was also involved in the generation of ROS (20).

Contemporarily with these works, Wang et al. analyzed which genes are potentially involved in regulating glucocorticoid-induced apoptosis in lymphoid cells. Through an oligonucleotide microarray analysis, they discovered a novel dexamethasone-induced gene designated *Dig2* whose expression is significantly induced in cell lines of murine T-cell lymphoma and in normal mouse thymocytes (21). The official name given by the HUGO Gene Nomenclature Committee was *DDIT4*.

*DDIT4* is located on chromosome 10 (10q22.1) and has a length of 2.1 kb, containing three exons and two introns (19, 20). *DDIT4* has three splice variants (one it is the protein coding with 232 amino acids and the others are retained introns), 95 orthologs, one paralog (*DDIT4L*, DNA damage inducible transcript 4 like) and is associated with one phenotype (22). Because of this, it is presumed that it was present in the common ancestor of animals (23).

**Figure 1:**
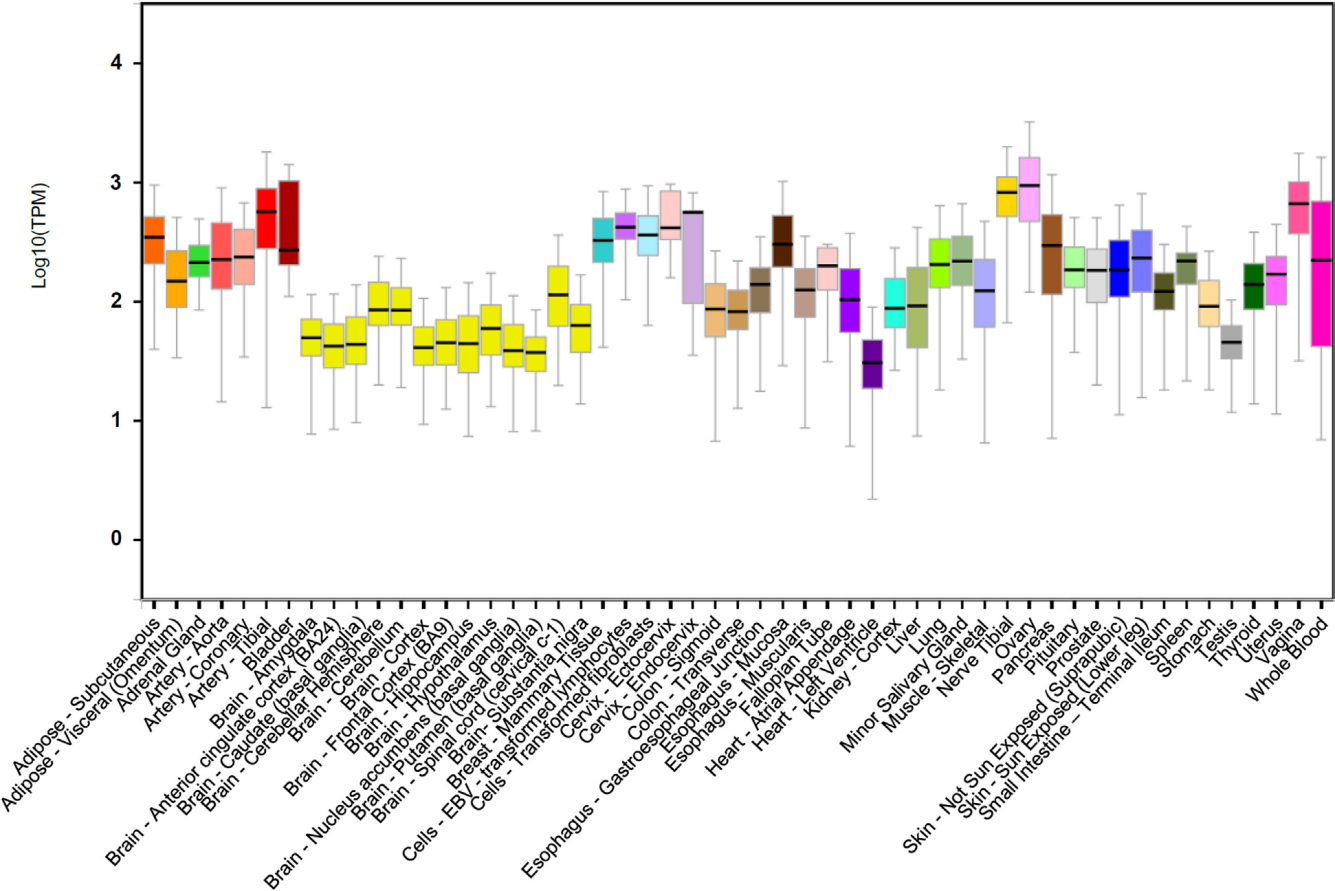
DNA damage inducible transcript 4 (*DDIT4*) gene expression. *DDIT4* is ubiquitously expressed in multiple human tissues (32).

*DDIT4* is ubiquitously expressed at low levels in most adult tissues (Figure 1) (19). The *DDIT4* expression is induced by multiple cellular stresses, such as hypoxia (19, 24), ionizing radiation (IR) (20), methyl methane sulfonate (MMS) (25), heat shock (21), and energy depletion (12). Moreover, it is also upregulated by other chemical molecules, such as glucocorticoids (21, 26, 27), dopaminergic neurotoxins (28), endoplasmic reticulum stress inducers (21, 29), DNA damage agent etoposide (21), and arsenite (30). Conversely, *DDIT4* expression decreases by testosterone, acute resistance exercise, refeeding/nutrient consumption, and suppressed mTORC1 (31).

## DDIT4 Protein

With an estimated half-life of approximately 5 min (33, 34), DDIT4 is a highly conserved protein composed of 232 amino acids, rich in leucine (17%) and contains conserved 9- or 10-serine stretches at its N-terminal end. Its molecular weight is 25 kDa, but it migrates around 35 kDa on Western blot because of its multiple lysine residues at the carboxyl terminus (19, 20). In addition, DDIT4 is mainly present in the cytoplasm and the nucleus (20, 30), but it has also been observed in membranes (35).

**Figure 2:**
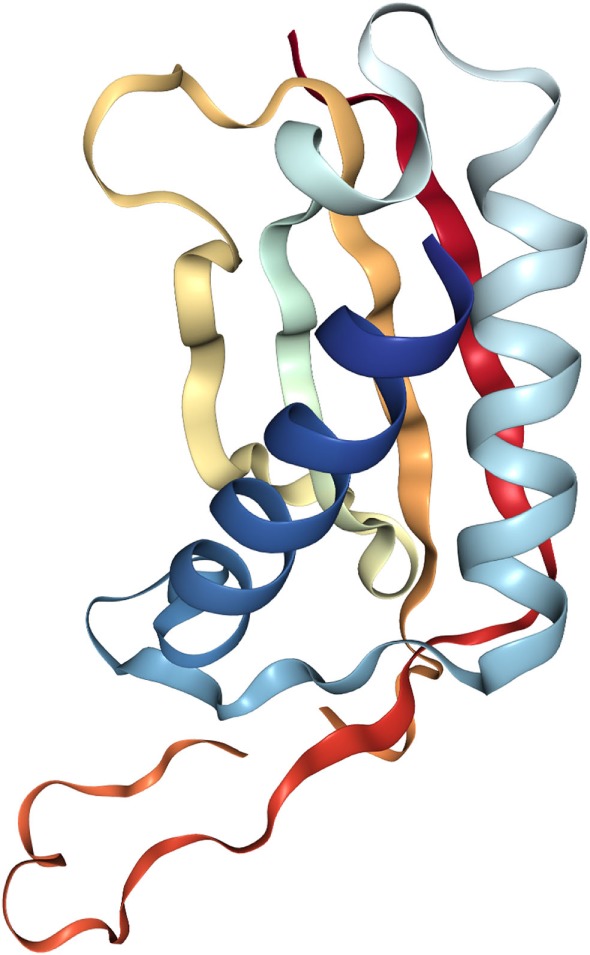
Crystal structure of the human protein DNA damage inducible transcript 4 (PDB ID# 3LQ9). Both of their chains have antiparallel α-helices followed by four β-strands.

The crystal structure of DDIT4 (PDB ID# 3LQ9) shows that this protein has two chains (A and B), where each chain has antiparallel α-helices followed by four β-strands with two highly conserved residues (residues 138–141 and 218–225) that might be essential for the activity of the protein (Figure 2). Amino acids in position 85–193 and 207–225 correspond to linear segments required for its function, separated by a dispensable region. The extreme N-terminus has 84 amino acids residues poorly preserved among species and dispensable for the function of the protein. By contrast, the C-terminal region is highly conserved and essential for the correct function of DDIT4. Internal deletions and NAAIRS (the sequence Asn–Ala–Ala–Ile–Arg–Ser) substitutions are poorly tolerated because this causes the protein to change to an unphysiological conformation (36).

## Regulation of DDIT4

*DDIT4* expression is upregulated by several transcription factors in response to different stressors (37). For example, under hypoxic conditions, HIF-1 increases the transcription of *DDIT4* by binding to a hypoxia response element of the *DDIT4* promoter causing downregulation of mTOR (19, 24). Telomere destabilization by DNA-damaging agents also increases *DDIT4* expression through multiple pathways. In mouse embryo fibroblasts (MEFs), IR induces *DDIT4* expression through p53 pathway in a dependent manner (20). Similarly, in HaCaT human keratinocytes, MMS induces *DDIT4* expression through Elk-1 pathway and CCAAT/enhancer-binding protein (C/EBP) but in a p53-independent manner (25). Another transcription factor is the nuclear factor of activated T-cells 3, whose overexpression increases c-Myc (mTOR downstream target) and MUC2 expression (a marker of goblet cell differentiation) through the induction of *DDIT4* expression; important process to the regulation of intestinal cell differentiation (38).

MicroRNAs, small and highly conserved non-coding RNA molecules, are also involved in tumorigenesis by regulating (at posttranscriptional level) specific oncogenes and tumor suppressor genes (39–41), such as *DDIT4*. miR-221 overexpression contributes to liver tumorigenesis through the cyclin-dependent kinase inhibitor p27 (Kip1-CDKN1B) and/or *DDIT4* downregulation (42). miR-495, directly upregulated by the transcription factors E12/E47 in breast cancer stem cells promotes oncogenesis and hypoxia resistance *via* downregulation of E-cadherin and *DDIT4* (43). miR-30c plays a key role in radiation-induced cell damage because, maybe in part, it downregulates *DDIT4* expression in human hematopoietic and osteoblast cells after gamma-irradiation (44). miR-630 has a bimodal role in the regulation of apoptosis in response to DNA damage; it promotes apoptosis by downregulation of cell cycle kinase 7 kinase, and on the other hand, it reduces apoptosis by downregulating apoptotic activators, such as DDIT4, PARP3, EP300, and p53 (45).

At posttranslational level, DDIT4 is quickly degraded by the ubiquitin-proteasome system to allow cells to restore mTOR signaling once the stress conditions have been mitigated. One of the models reported by Katiyar et al. consists in the phosphorylation of DDIT4 by GSK3-β, which causes the recruitment of the Cullin 4A (CUL4A)–DNA damage-binding protein 1–regulator of cullins 1–β-transducin repeat containing protein (β-TRCP) E3 ligase complex, that results in DDIT4 ubiquitination and degradation by the proteasome (34). Amplification and overexpression of *CUL4A* have been observed in primary breast cancers (46) and others types of cancers such as hepatocellular carcinomas (47), so it could be considered as a potential predictive and prognostic indicator of some cancers. In addition, increased β-TRCP mRNA and protein expression have also been found in colorectal and pancreatic cancers (48, 49). By contrast, Tan and Hagen reported that there is an alternative E3 ligase (currently unknown) responsible for both basal DDIT4 ubiquitination and ubiquitination that is induced upon mTORC1 inhibition (50). These processes sustain that the dysregulation of DDIT4 degradation could be a common event that elevates mTOR signaling during tumor development.

## *DDIT4* Inhibits the Activity of mTOR *VIA* Tuberous Sclerosis Complex (TSC1/TSC2 Complex)

All the stressors mentioned above, *via* different transcription factors, elevate the *DDIT4* expression to fulfill its main function, inhibit mTORC1 to regulate key cellular processes, such as cell growing, proliferation, and survival (12, 24). This inhibitory effect was initially identified in *Drosophila*, where expression of *Scylla* (homologous protein of DDIT4 in *Drosophila*) suppress the phosphorylation of S6K (a known substrate of TOR) (51); a similar process that was later confirmed in mammalian cells (52). In addition, TSC1/TSC2 complex does not interact directly with mTORC1, but it functions as a GTPase, inactivating to Rheb, converting Rheb-GTP into Rheb-GDP, unable to activate the mTORC1 complex (53).

**Figure 3:**
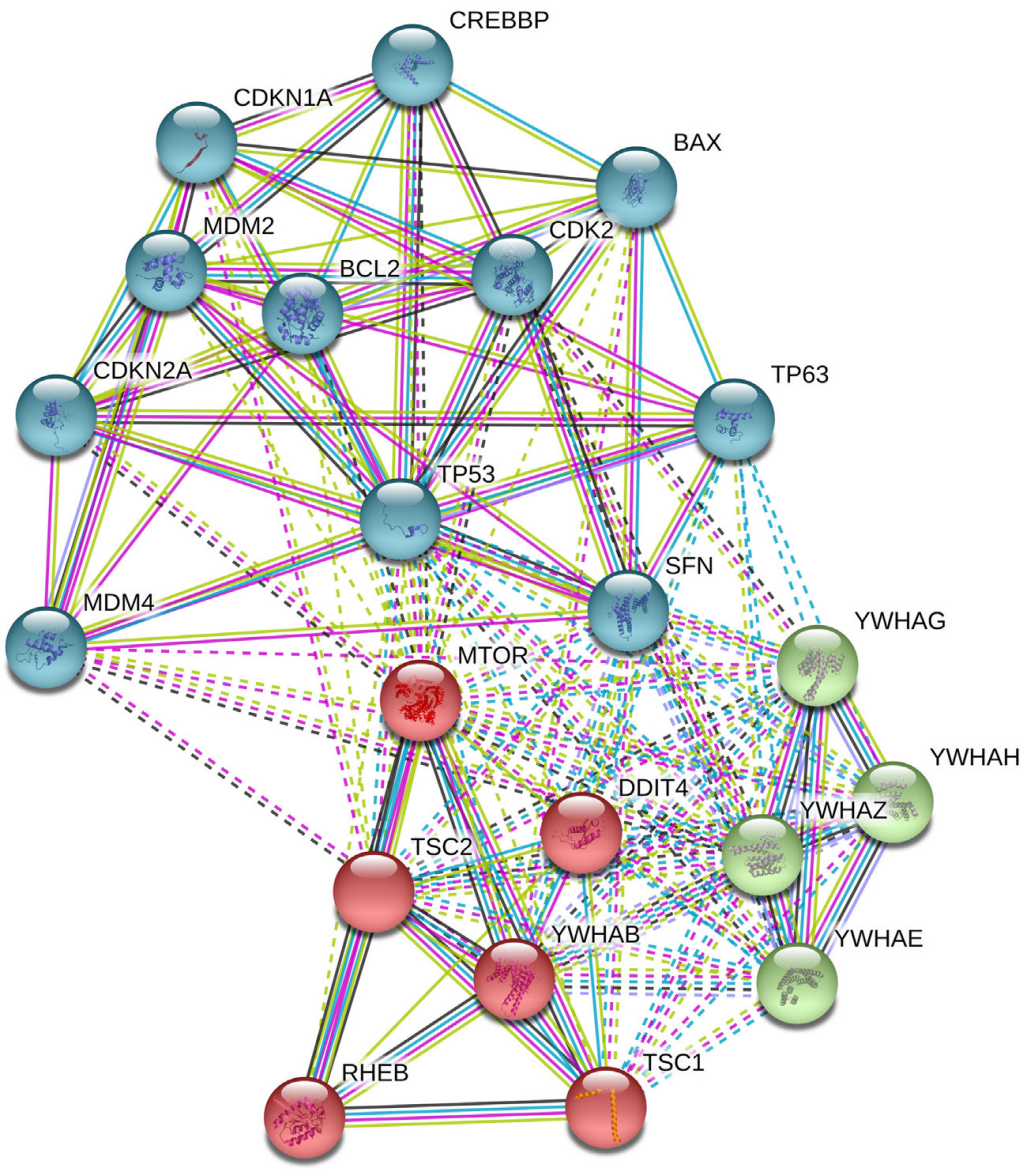
Analysis in STRING-DB v.10.5 describes that DNA damage inducible transcript 4 (DDIT4) is involved in the regulation of at least three clusters of proteins, (i) mammalian target of rapamycin (mTOR) pathway proteins (in red), (ii) p53 pathway (in sky blue), and (iii) 14-3-3 proteins (in green).

**Figure 4:**
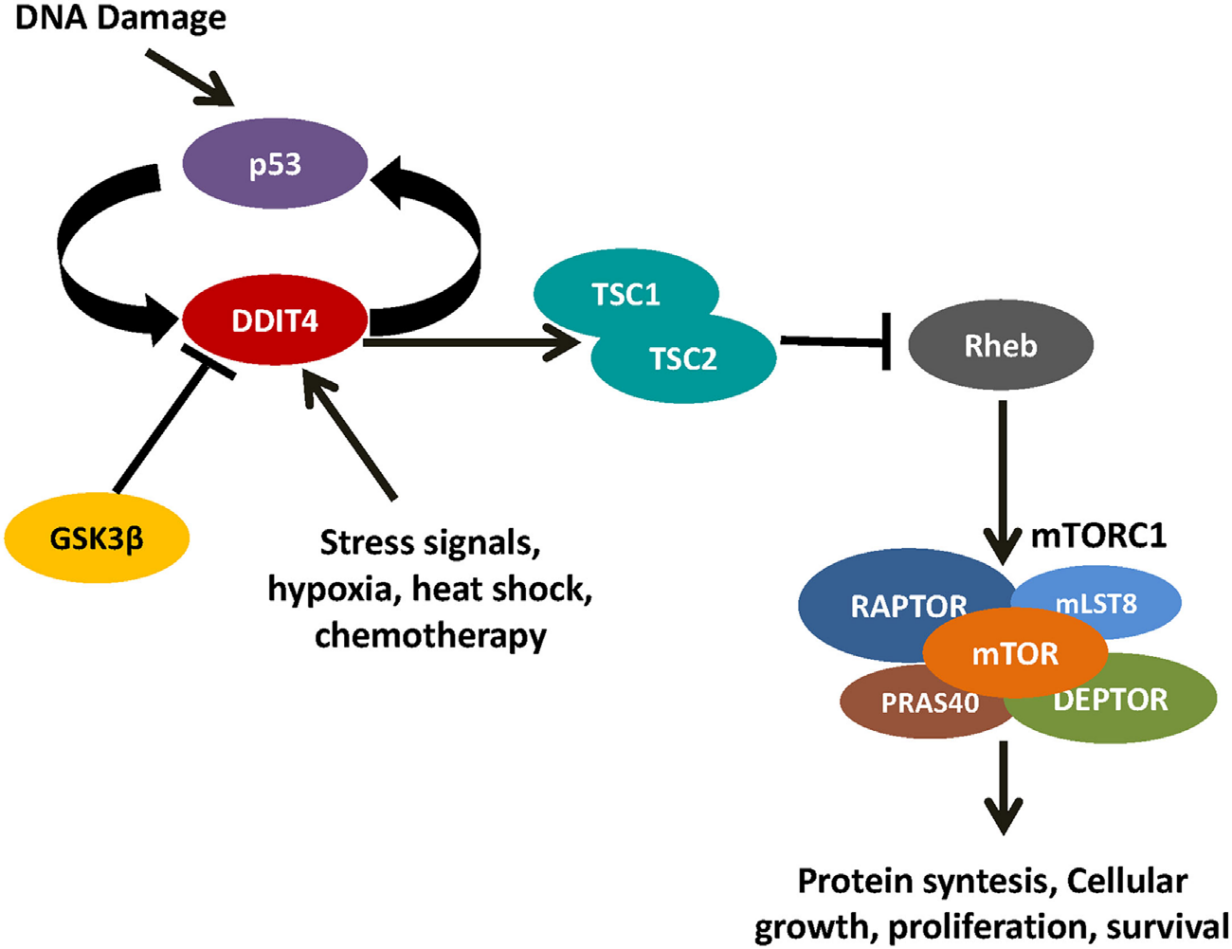
DNA damage inducible transcript 4 (DDIT4) controls mammalian target of rapamycin (mTOR) by the activation of the TSC1/TSC2 complex. When the TSC1/TSC2 complex inactivates Rheb, it is unable to activate mTOR complex 1.

Gordon et al. proposed two models of the effect of DDIT4 in the mTOR pathway. One model proposes that DDIT4 competes with TSC2 to bind with the 14-3-3 proteins. DDIT4 expression increases causing the dissociation of the 14-3-3 proteins with TSC2, so TSC2 is released to form a functional TSC1/TSC2 complex that inhibits mTORC1 activity (31, 35). However, functional and structural analysis has concluded that it is unlikely that DDIT4 interacts directly with 14-3-3 proteins, discarding this model (36). The other model proposes that phosphatase-2A recruit to Akt causing the reduction of Akt phosphorylation, which in turn causes the reduction of phosphorylation of TSC2 and its induction. The TSC1/TSC2 complex is formed and subsequently represses mTORC1 activity. By contrast, TSC1/TSC2 complex positively regulates mTORC2 through association with rapamycin-insensitive companion of mTOR (31) (Figures 3 and 4).

## DDIT4 and Autophagy

Autophagy is a catabolic mechanism of cellular adaptation to nutrients deprivation in which cellular components are degraded to its elementary molecules and recycled to promote the cell survival (54). In addition, autophagy participates in the removal of old or damaged cellular organelles and has been associated with the survival of cancer cells (55). Autophagy is the opposite response to cell growth and proliferation induced by mTOR activity. Under favorable nutritional conditions, the complex mTORC1 represses autophagy by phosphorylation of the ULK protein complex turning it in an open structural conformation (inactive form). DDIT4-mediated mTOR repression produces the lack of phosphorylation of the ULK complex, turning the complex in a closed structural conformation (active form) activating the mechanism of autophagy and triggering the formation of the autophagosome (56).

## Role of *DDIT4* in Cancer

Dysfunction of *DDIT4* has been associated with multiple diseases, such as neurodegenerative disorders (28, 57), ischemic proliferative retinopathy (58), preeclampsia (59), diabetes, and other degenerative pathologies, such as cancer (10). Results of *in vitro* studies suggest that *DDIT4* could have a context-dependent contrasting role in cancer as oncogene or tumor suppressor (19, 60).

Hyperactivation of the PI3K-Akt-mTOR signaling is a common alteration in cancer. This pathway regulates cellular processes involved in cell growth, proliferation, motility, survival, and apoptosis (61). In normal cells, PI3K-Akt-mTOR signaling is controlled by PTEN through PI3K dephosphorylation ensuring a transient and controlled activation. On the other hand, PTEN inactivation causes a chronic activation of PI3K and its downstream effectors, such as Akt, promoting the cell cycle progression, survival, and decreased apoptosis (62).

To know which genes are involved in the growth of cancer cells *via* chronically activated PIK-3 (such as in *PTEN^−/−^* cells), Schwarzer et al. compared *PIK3* expression between cells that have active *PIK-3* versus cells in which it is silenced. They found that *DDIT4* mRNA was significantly downregulated in prostate cancer cells (PC-3) using LY294002 (2-morpholin-4-yl-8-phenylchromen-4-one) or rapamycin, both inhibitors of PI3K, in combination with matrigel-based 3D culture system. By contrast, *DDIT4* was overexpressed under hypoxic conditions in a HIF-1α dependent manner. They also verified their results with other methods, as, for example, inhibiting the function of *DDIT4* employing antisense molecules or interference RNA, which indicates that DDIT4 is a transcriptional downstream target in the PI3K pathway and essential for invasive growth of PC-3 both *in vitro* and *in vivo* (13).

On the other hand, *DDIT4* has a key role in RAS signaling to transform human ovarian epithelial cells. *DDIT4* is overexpressed in RAS-transformed human ovarian epithelial cells lines T29 and T80 promoting cell proliferation and colony formation. *DDIT4* is overexpressed after activation of *RAS* oncogene, increasing levels of anti-apoptotic proteins and at the same time decreasing expression of pro-apoptotic proteins (60, 63).

Several works have described an involvement of *DDIT4* in the breast cancer biology, while its expression seems to have different patterns among breast cancer subtypes. Koo and Jung characterized the expression of proteins involved in mTOR and hypoxia pathway with staining with immunohistochemistry where immunophenotypes of breast cancer were determined by the evaluation of estrogen and progesterone receptors and HER2 while breast papilloma samples were included as controls (64).

In this study, Glut-1 and HIF-1α had higher expression in triple-negative breast cancer (TNBC) and HER2 phenotypes than in the luminal A and B phenotypes. These findings could be explained by the greater hypoxia conditions present in TNBC and HER2 tumors. Likewise, Ki-67 expression in TNBC and HER2 was higher than in other phenotypes or papilloma. Interestingly, downregulation of DDIT4 does not lead to a negative feedback to HIF-1 α, so the tumorigenesis mediated by HIF-1α is constant. Moreover, this study demonstrated that HIF-1α expression was associated with reduced disease-free survival (DFS) and reduced overall survival (OS), concordant with previous studies (65–67). Concluding that TNBC and HER2 overexpression showed the highest cell proliferation and survival in a hypoxic tumor environment by activation of the mTOR pathway and HIF-1α stabilization *via* DDIT4 downregulation (64).

*DDIT4* participate in an endogenous feedback circuit with p53. When cells and tissues of *DDIT4*^−/−^ mice are exposed to IR and chemotherapy treatment, the genetic loss of *DDIT4* confers an increase in DNA damage-induced apoptosis both *in vitro* and *in vivo*, which is associated with elevated levels of p53 protein following DNA damage. It was proved that deregulation of p53 is not due to increased p53 stabilization or failed DNA repair but, instead, to increased p53 translation. Consistent with this, it was demonstrated that *DDIT4* loss elevates the mTORC1 activity, which explains the increased p53 translation and damage sensitivity in *DDIT4*^−/−^ cells (68).

Under stress conditions mTORC1 is also inhibited by the activation of p53 both dependently and independently of Sertrin1/2, suggesting the existence of more than one pathway to inhibit mTORC1 (69–71). To test this, Cam et al. exposed MEFs and *in vivo* tumor models to drugs that induce DNA damage to analyze the upstream regulation of mTORC1 signaling. They found that inhibition of mTORC1 signaling to 4EBP1 requires the coordinated activity of both p53 and p63; by contrast, the inhibition of S6K1 and rpS6 phosphorylation is Akt-dependent. Concordant with this, the loss of p53 or p63 prevents the suppression of mTORC1 signaling induced by the DNA damage, supporting that both are necessary for the inhibition of mTORC1. Suggesting that there are multiples mechanisms that suppress p53/p63 responses and at the same time suppress the ability of the cancer cell to control mTORC1 (72).

In response to DNA damage, DDIT4 phosphorylates downstream Akt through the TSC1/TSC2 complex to inhibit mTORC1 signaling (73, 74). To investigate the clinical significance of this process, Wei et al. analyzed the DDIT4 and p-Akt expression in ovary cancer (primary ovarian cancer and borderline tumors) and normal fallopian tubes. Both DDIT4 and p-Akt expressions were significantly higher in patients with serous ovarian cancer and late FIGO stage; while only DDIT4 expression was significantly higher in ascites formation and only p-Akt expression was significantly histological grade and chemoresistance. Features of patients with improved outcomes in terms of DFS and OS were low DDIT4 staining or absence of p-Akt. In this work, there was no association between *KRAS* mutations and DDIT4 intensity staining, suggesting that *KRAS* is not involved in the activation of DDIT4 (15).

In contrast to the study performed by Koo and Jung, where they reported that *DDIT4* is downregulated in TNBC and HER2 overexpression types (64), Pinto et al. found that *DDIT4* is associated with poor prognosis in TNBC resistant to neoadjuvant chemotherapy (17).

All these data presented previously show that *DDIT4* has a key role in different types of cancer and its aggressiveness. A recent *in silico* analysis of *DDIT4* expression in several cancer types showed that the high expression of this gene was related to a bad outcome in diverse hematologic and solid tumors, such as acute myeloid leukemia, breast cancer, glioblastoma multiforme, melanoma, lung, and colon cancer. Furthermore, it was shown that response to rapamycin and others mTOR inhibitors were not influenced by *DDIT4* expression (18).

## Concluding Remarks and Future Perspectives

The increasing evidence about the involvement of *DDIT4* in key cellular mechanisms of tumor aggressiveness suggests its driver role in cancer and consequently, its potential not only as prognostic biomarker but also as a therapeutic target. Since mTOR inhibitors have shown disappointing results in the treatment of cancer, where the main issues are the lack of a biomarker to select patients who will benefit from these drugs and the poor knowledge about mechanisms of resistance, a better therapeutic strategy would be *DDIT4* inhibition. In contrast to mTOR expression, the high *DDIT4* expression is related with a poor outcome. We hypothesize DDIT4 targeting could lead to cancer cells to avoid the metabolic suppression needed for cell survival under stress conditions (e.g., treatment with cytotoxic chemotherapy or radiotherapy). Combination of DDIT4 inhibitors with DNA-damaging agents, such as cisplatin, will be interesting, especially in tumors with loss of function of *TP53*, because wild-type p53 repress the expression of DDIT4 in a regulatory loop, potentiating the effects of DDIT4 inhibitors.

## Author Contributions

IT-H gathered all the information and wrote the first draft of the manuscript. WF wrote, corrected, and also provided important contributions to the final manuscript. JP contributed to the conception and design of the project, critically reviewing the entire manuscript. All authors contributed to manuscript revision, read and approved the submitted version.

## Conflict of Interest Statement

The authors declare that the research was conducted in the absence of any commercial or financial relationships that could be construed as a potential conflict of interest.

## References

[1] Hanahan D Weinberg RA The hallmarks of cancer. Cell (2000) 100(1):57–70. 10.1016/S0092-8674(00)81683-9 10647931

[2] Bianco R Melisi D Ciardiello F Tortora G. Key cancer cell signal transduction pathways as therapeutic targets. Eur J Cancer (2006) 42(3):290–4. 10.1016/j.ejca.2005.07.034 16376541

[3] Maiese K Therapeutic targets for cancer: current concepts with PI 3-K, Akt, & mTOR. Indian J Med Res (2013) 137(2):243–6. 23563366 PMC3657846

[4] Mita MM Mita A Rowinsky EK. The molecular target of rapamycin (mTOR) as a therapeutic target against cancer. Cancer Biol Ther (2003) 2(4 Suppl 1):S169–77. 10.4161/cbt.365 14508096

[5] Laplante M Sabatini DM. mTOR signaling in growth control and disease. Cell (2012) 149(2):274–93. 10.1016/j.cell.2012.03.017 22500797 PMC3331679

[6] Wullschleger S Loewith R Hall MN TOR signaling in growth and metabolism. Cell (2006) 124(3):471–84. 10.1016/j.cell.2006.01.016 16469695

[7] Hay N Sonenberg N. Upstream and downstream of mTOR. Genes Dev (2004) 18(16):1926–45. 10.1101/gad.1212704 15314020

[8] Loewith R Jacinto E Wullschleger S Lorberg A Crespo JL Bonenfant D Two TOR complexes, only one of which is rapamycin sensitive, have distinct roles in cell growth control. Mol Cell (2002) 10(3):457–68. 10.1016/S1097-2765(02)00636-6 12408816

[9] Pópulo H Lopes JM Soares P. The mTOR signalling pathway in human cancer. Int J Mol Sci (2012) 13(2):1886–918. 10.3390/ijms13021886 22408430 PMC3291999

[10] Zoncu R Efeyan A Sabatini DM. mTOR: from growth signal integration to cancer, diabetes and ageing. Nat Rev Mol Cell Biol (2011) 12(1):21–35. 10.1038/nrm3025 21157483 PMC3390257

[11] Thoreen CC Sabatini DM Rapamycin inhibits mTORC1, but not completely. Autophagy (2009) 5(5):725–6. 10.4161/auto.5.5.8504 19395872

[12] Sofer A Lei K Johannessen CM Ellisen LW. Regulation of mTOR and cell growth in response to energy stress by REDD1. Mol Cell Biol (2005) 25(14):5834–45. 10.1128/MCB.25.14.5834-5845.2005 15988001 PMC1168803

[13] Schwarzer R Tondera D Arnold W Giese K Klippel A Kaufmann J. REDD1 integrates hypoxia-mediated survival signaling downstream of phosphatidylinositol 3-kinase. Oncogene (2005) 24(7):1138–49. 10.1038/sj.onc.1208236 15592522

[14] An SJ Chen JK Liu LL Zhao YF Chen XM. Over-expressed genes detected by suppression subtractive hybridization in carcinoma derived from transformed 16HBE cells induced by BPDE. Biomed Environ Sci (2005) 18(5):302–6. 16370312

[15] Jia W Chang B Sun L Zhu H Pang L Tao L REDD1 and p-AKT over-expression may predict poor prognosis in ovarian cancer. Int J Clin Exp Pathol (2014) 7(9):5940–9. 25337238 PMC4203209

[16] Çelik H Bulut G Han J Graham GT Minas TZ Conn EJ Ezrin inhibition up-regulates stress response gene expression. J Biol Chem (2016) 291(25):13257–70. 10.1074/jbc.M116.718189 27137931 PMC4933238

[17] Pinto JA Araujo J Cardenas NK Morante Z Doimi F Vidaurre T A prognostic signature based on three-genes expression in triple-negative breast tumours with residual disease. NPJ Genom Med (2016) 1:15015. 10.1038/npjgenmed.2015.15 29263808 PMC5685288

[18] Pinto JA Rolfo C Raez LE Prado A Araujo JM Bravo L In silico evaluation of DNA damage inducible transcript 4 gene (DDIT4) as prognostic biomarker in several malignancies. Sci Rep (2017) 7(1):1526. 10.1038/s41598-017-01207-3 28484222 PMC5431475

[19] Shoshani T Faerman A Mett I Zelin E Tenne T Gorodin S Identification of a novel hypoxia-inducible factor 1-responsive gene, RTP801, involved in apoptosis. Mol Cell Biol (2002) 22(7):2283–93. 10.1128/MCB.22.7.2283-2293.2002 11884613 PMC133671

[20] Ellisen LW Ramsayer KD Johannessen CM Yang A Beppu H Minda K REDD1, a developmentally regulated transcriptional target of p63 and p53, links p63 to regulation of reactive oxygen species. Mol Cell (2002) 10(5):995–1005. 10.1016/S1097-2765(02)00706-2 12453409

[21] Wang Z Malone MH Thomenius MJ Zhong F Xu F Distelhorst CW. Dexamethasone-induced gene 2 (dig2) is a novel pro-survival stress gene induced rapidly by diverse apoptotic signals. J Biol Chem (2003) 278(29):27053–8. 10.1074/jbc.M303723200 12736248

[22] Gene: DDIT4 (ENSG00000168209) – Summary – Homo sapiens – Ensembl Genome Browser 91 [Internet]. (2017). Available from: http://www.ensembl.org/Homo_sapiens/Gene/Summary?g=ENSG00000168209;r=10:72273920-72276036 (Accessed: February 19, 2018).

[23] Gene: DDIT4 (ENSG00000168209) – Gene Tree – Homo sapiens – Ensembl Genome Browser 90 [Internet]. (2017). Available from: http://www.ensembl.org/Homo_sapiens/Gene/Compara_Tree?db=core;g=ENSG00000168209;r=10:72273920-72276036 (Accessed: October 9, 2017).

[24] Brugarolas J Lei K Hurley RL Manning BD Reiling JH Hafen E Regulation of mTOR function in response to hypoxia by REDD1 and the TSC1/TSC2 tumor suppressor complex. Genes Dev (2004) 18(23):2893–904. 10.1101/gad.1256804 15545625 PMC534650

[25] Lin L Qian Y Shi X Chen Y. Induction of a cell stress response gene RTP801 by DNA damaging agent methyl methanesulfonate through CCAAT/enhancer binding protein. Biochemistry (Mosc) (2005) 44(10):3909–14. 10.1021/bi047574r 15751966

[26] Molitoris JK McColl KS Swerdlow S Matsuyama M Lam M Finkel TH Glucocorticoid elevation of dexamethasone-induced gene 2 (Dig2/RTP801/REDD1) protein mediates autophagy in lymphocytes. J Biol Chem (2011) 286(34):30181–9. 10.1074/jbc.M111.245423 21733849 PMC3191057

[27] Wolff NC McKay RM Brugarolas J. REDD1/DDIT4-independent mTORC1 inhibition and apoptosis by glucocorticoids in thymocytes. Mol Cancer Res (2014) 12(6):867–77. 10.1158/1541-7786.MCR-13-0625 24615339 PMC4260655

[28] Malagelada C López-Toledano MA Willett RT Jin ZH Shelanski ML Greene LA. RTP801/REDD1 regulates the timing of cortical neurogenesis and neuron migration. J Neurosci (2011) 31(9):3186–96. 10.1523/JNEUROSCI.4011-10.2011 21368030 PMC3089438

[29] Whitney ML Jefferson LS Kimball SR. ATF4 is necessary and sufficient for ER stress-induced upregulation of REDD1 expression. Biochem Biophys Res Commun (2009) 379(2):451–5. 10.1016/j.bbrc.2008.12.079 19114033 PMC2656673

[30] Lin L Stringfield TM Shi X Chen Y. Arsenite induces a cell stress-response gene, RTP801, through reactive oxygen species and transcription factors Elk-1 and CCAAT/enhancer-binding protein. Biochem J (2005) 392(Pt 1):93–102. 10.1042/BJ20050553 16008523 PMC1317668

[31] Gordon BS Steiner JL Williamson DL Lang CH Kimball SR. Emerging role for regulated in development and DNA damage 1 (REDD1) in the regulation of skeletal muscle metabolism. Am J Physiol (2016) 311(1):E157–74. 10.1152/ajpendo.00059.2016 27189933 PMC4967146

[32] GTEx Consortium, Laboratory, Data Analysis &Coordinating Center (LDACC)—Analysis Working Group, Statistical Methods Groups—Analysis Working Group, Enhancing GTEx (eGTEx) Groups, NIH Common Fund, NIH/NCI, NIH/NIMH Genetic effects on gene expression across human tissues. Nature (2017) 550(7675):204–13. 10.1038/nature24277 29022597 PMC5776756

[33] Kimball SR Do AND Kutzler L Cavener DR Jefferson LS. Rapid turnover of the mTOR complex 1 (mTORC1) repressor REDD1 and activation of mTORC1 signaling following inhibition of protein synthesis. J Biol Chem (2008) 283(6):3465–75. 10.1074/jbc.M706643200 18070882 PMC2654224

[34] Katiyar S Liu E Knutzen CA Lang ES Lombardo CR Sankar S REDD1, an inhibitor of mTOR signalling, is regulated by the CUL4A–DDB1 ubiquitin ligase. EMBO Rep (2009) 10(8):866–72. 10.1038/embor.2009.93 19557001 PMC2726664

[35] DeYoung MP Horak P Sofer A Sgroi D Ellisen LW Hypoxia regulates TSC1/2–mTOR signaling and tumor suppression through REDD1-mediated 14–3–3 shuttling. Genes Dev (2008) 22(2):239–51. 10.1101/gad.1617608 18198340 PMC2192757

[36] Vega-Rubin-de-Celis S Abdallah Z Kinch L Grishin NV Brugarolas J Zhang X. Structural analysis and functional implications of the negative mTORC1 regulator REDD1. Biochemistry (Mosc) (2010) 49(11):2491–501. 10.1021/bi902135e 20166753 PMC3046781

[37] Canal M Romaní-Aumedes J Martín-Flores N Pérez-Fernández V Malagelada C. RTP801/REDD1: a stress coping regulator that turns into a troublemaker in neurodegenerative disorders. Front Cell Neurosci (2014) 8:313. 10.3389/fncel.2014.00313 25324725 PMC4183088

[38] Zhou Y Wang Q Guo Z Weiss HL Evers BM. Nuclear factor of activated T-cell c3 inhibition of mammalian target of rapamycin signaling through induction of regulated in development and DNA damage response 1 in human intestinal cells. Mol Biol Cell (2012) 23(15):2963–72. 10.1091/mbc.E12-01-0037 22696685 PMC3408422

[39] MacFarlane L-A Murphy PR MicroRNA: biogenesis, function and role in cancer. Curr Genomics (2010) 11(7):537–61. 10.2174/138920210793175895 21532838 PMC3048316

[40] Calin GA Croce CM. MicroRNA signatures in human cancers. Nat Rev Cancer (2006) 6(11):857–66. 10.1038/nrc1997 17060945

[41] Iorio MV Croce CM. MicroRNAs in cancer: small molecules with a huge impact. J Clin Oncol (2009) 27(34):5848–56. 10.1200/JCO.2009.24.0317 19884536 PMC2793003

[42] Pineau P Volinia S McJunkin K Marchio A Battiston C Terris B miR-221 overexpression contributes to liver tumorigenesis. Proc Natl Acad Sci U S A (2010) 107(1):264–9. 10.1073/pnas.0907904107 20018759 PMC2806773

[43] Hwang-Verslues WW Chang P-H Wei P-C Yang C-Y Huang C-K Kuo W-H miR-495 is upregulated by E12/E47 in breast cancer stem cells, and promotes oncogenesis and hypoxia resistance via downregulation of E-cadherin and REDD1. Oncogene (2011) 30(21):2463–74. 10.1038/onc.2010.618 21258409

[44] Li XH Ha CT Fu D Xiao M. Micro-RNA30c negatively regulates REDD1 expression in human hematopoietic and osteoblast cells after gamma-irradiation. PLoS One (2012) 7(11):e48700. 10.1371/journal.pone.0048700 23144934 PMC3492427

[45] Cao J-X Lu Y Qi J-J An G-S Mao Z-B Jia H-T MiR-630 inhibits proliferation by targeting CDC7 kinase, but maintains the apoptotic balance by targeting multiple modulators in human lung cancer A549 cells. Cell Death Dis (2014) 5:e1426. 10.1038/cddis.2014.386 25255219 PMC4225225

[46] Chen LC Manjeshwar S Lu Y Moore D Ljung BM Kuo WL The human homologue for the *Caenorhabditis elegans* cul-4 gene is amplified and overexpressed in primary breast cancers. Cancer Res (1998) 58(16):3677–83. 9721878

[47] Yasui K Arii S Zhao C Imoto I Ueda M Nagai H TFDP1, CUL4A, and CDC16 identified as targets for amplification at 13q34 in hepatocellular carcinomas. Hepatology (2002) 35(6):1476–84. 10.1053/jhep.2002.33683 12029633

[48] Müerköster S Arlt A Sipos B Witt M Grossmann M Klöppel G Increased expression of the E3-ubiquitin ligase receptor subunit betaTRCP1 relates to constitutive nuclear factor-kappaB activation and chemoresistance in pancreatic carcinoma cells. Cancer Res (2005) 65(4):1316–24. 10.1158/0008-5472.CAN-04-1626 15735017

[49] Ougolkov A Zhang B Yamashita K Bilim V Mai M Fuchs SY Associations among beta-TrCP, an E3 ubiquitin ligase receptor, beta-catenin, and NF-kappaB in colorectal cancer. J Natl Cancer Inst (2004) 96(15):1161–70. 10.1093/jnci/djh219 15292388

[50] Tan CY Hagen T. mTORC1 dependent regulation of REDD1 protein stability. PLoS One (2013) 8(5):e63970. 10.1371/journal.pone.0063970 23717519 PMC3661664

[51] Reiling JH Hafen E. The hypoxia-induced paralogs *Scylla* and *Charybdis* inhibit growth by down-regulating S6K activity upstream of TSC in *Drosophila*. Genes Dev (2004) 18(23):2879–92. 10.1101/gad.322704 15545626 PMC534649

[52] Corradetti MN Inoki K Guan K-L. The stress-inducted proteins RTP801 and RTP801L are negative regulators of the mammalian target of rapamycin pathway. J Biol Chem (2005) 280(11):9769–72. 10.1074/jbc.C400557200 15632201

[53] Long X Lin Y Ortiz-Vega S Yonezawa K Avruch J. Rheb binds and regulates the mTOR kinase. Curr Biol (2005) 15(8):702–13. 10.1016/j.cub.2005.02.053 15854902

[54] Mariño G Niso-Santano M Baehrecke EH Kroemer G. Self-consumption: the interplay of autophagy and apoptosis. Nat Rev Mol Cell Biol (2014) 15(2):81–94. 10.1038/nrm3735 24401948 PMC3970201

[55] Kimmelman AC The dynamic nature of autophagy in cancer. Genes Dev (2011) 25(19):1999–2010. 10.1101/gad.17558811 21979913 PMC3197199

[56] Jung CH Ro S-H Cao J Otto NM Kim D-H. mTOR regulation of autophagy. FEBS Lett (2010) 584(7):1287–95. 10.1016/j.febslet.2010.01.017 20083114 PMC2846630

[57] Kim J-R Lee S-R Chung HJ Kim S Baek S-H Kim JH Identification of amyloid beta-peptide responsive genes by cDNA microarray technology: involvement of RTP801 in amyloid beta-peptide toxicity. Exp Mol Med (2003) 35(5):403–11. 10.1038/emm.2003.53 14646594

[58] Brafman A Mett I Shafir M Gottlieb H Damari G Gozlan-Kelner S Inhibition of oxygen-induced retinopathy in RTP801-deficient mice. Invest Ophthalmol Vis Sci (2004) 45(10):3796–805. 10.1167/iovs.04-0052 15452091

[59] Hu YY Liu JC Xing AY You Y Wang XD. REDD1 expression in placenta during human gestation. Reprod Sci (2012) 19(9):995–1000. 10.1177/1933719112440054 22527987

[60] Chang B Liu G Yang G Mercado-Uribe I Huang M Liu J. REDD1 is required for RAS-mediated transformation of human ovarian epithelial cells. Cell Cycle (2009) 8(5):780–6. 10.4161/cc.8.5.7887 19221489

[61] Porta C Paglino C Mosca A Targeting PI3K/Akt/mTOR signaling in cancer. Front Oncol (2014) 4:64 10.3389/fonc.2014.00064 24782981 PMC3995050

[62] Khan KH Yap TA Yan L Cunningham D. Targeting the PI3K-AKT-mTOR signaling network in cancer. Chin J Cancer (2013) 32(5):253–65. 10.5732/cjc.013.10057 23642907 PMC3845556

[63] Smith ER Xu X-X REDD1, a new Ras oncogenic effector. Cell Cycle (2009) 8(5):675–6. 10.4161/cc.8.5.8184 19242117

[64] Koo JS Jung W Alteration of REDD1-mediated mammalian target of rapamycin pathway and hypoxia-inducible factor-1α regulation in human breast cancer. Pathobiology (2010) 77(6):289–300. 10.1159/000320936 21266827

[65] Bos R van der Groep P Greijer AE Shvarts A Meijer S Pinedo HM Levels of hypoxia-inducible factor-1alpha independently predict prognosis in patients with lymph node negative breast carcinoma. Cancer (2003) 97(6):1573–81. 10.1002/cncr.11246 12627523

[66] Schindl M Schoppmann SF Samonigg H Hausmaninger H Kwasny W Gnant M Overexpression of hypoxia-inducible factor 1alpha is associated with an unfavorable prognosis in lymph node-positive breast cancer. Clin Cancer Res (2002) 8(6):1831–7. 12060624

[67] Trastour C Benizri E Ettore F Ramaioli A Chamorey E Pouysségur J HIF-1α and CA IX staining in invasive breast carcinomas: prognosis and treatment outcome. Int J Cancer (2007) 120(7):1451–8. 10.1002/ijc.22436 17245699

[68] Vadysirisack DD Baenke F Ory B Lei K Ellisen LW. Feedback control of p53 translation by REDD1 and mTORC1 limits the p53-dependent DNA damage response. Mol Cell Biol (2011) 31(21):4356–65. 10.1128/MCB.05541-11 21896779 PMC3209328

[69] Budanov AV Karin M. p53 target genes sestrin1 and sestrin2 connect genotoxic stress and mTOR signaling. Cell (2008) 134(3):451–60. 10.1016/j.cell.2008.06.028 18692468 PMC2758522

[70] Matthew EM Hart LS Astrinidis A Navaraj A Dolloff NG Dicker DT The p53 target Plk2 interacts with TSC proteins impacting mTOR signaling, tumor growth and chemosensitivity under hypoxic conditions. Cell Cycle (2009) 8(24):4168–75. 10.4161/cc.8.24.10800 20054236 PMC2975271

[71] Feng Z Hu W de Stanchina E Teresky AK Jin S Lowe S The regulation of AMPK beta1, TSC2, and PTEN expression by p53: stress, cell and tissue specificity, and the role of these gene products in modulating the IGF-1-AKT-mTOR pathways. Cancer Res (2007) 67(7):3043–53. 10.1158/0008-5472.CAN-06-4149 17409411

[72] Cam M Bid HK Xiao L Zambetti GP Houghton PJ Cam H. p53/TAp63 and AKT regulate mammalian target of rapamycin complex 1 (mTORC1) signaling through two independent parallel pathways in the presence of DNA damage. J Biol Chem (2014) 289(7):4083–94. 10.1074/jbc.M113.530303 24366874 PMC3924274

[73] Dennis MD McGhee NK Jefferson LS Kimball SR. Regulated in DNA damage and development 1 (REDD1) promotes cell survival during serum deprivation by sustaining repression of signaling through the mechanistic target of rapamycin in complex 1 (mTORC1). Cell Signal (2013) 25(12):2709–16. 10.1016/j.cellsig.2013.08.038 24018049 PMC3867791

[74] Jin H-O Hong S-E Kim J-H Choi H-N Kim K An S Sustained overexpression of REDD1 leads to Akt activation involved in cell survival. Cancer Lett (2013) 336(2):319–24. 10.1016/j.canlet.2013.03.021 23528835

